# Failure pattern and suggestions for target volume delineation of carcinoma showing thymus-like differentiation treated with intensity-modulated radiotherapy

**DOI:** 10.1186/s12885-022-10171-9

**Published:** 2022-10-21

**Authors:** Fang-Fang Kong, Guang-Sen Pan, Rui-Ping Zhai, Cheng-Run Du, Xia-Yun He, Chun-Ying Shen, Xue-Guan Lu, Tuan-Qi Sun, Yu Wang, Qing-Hai Ji, Chao-Su Hu, Hong-Mei Ying

**Affiliations:** 1 Department of Radiation Oncology, Fudan University Shanghai Cancer Center, 200032 Shanghai, China; 2 Department of Oncology, Shanghai Medical College, Fudan University, 200032 Shanghai, China; 3 Department of Head and Neck Surgery, Fudan University Shanghai Cancer Center, 200032 Shanghai, China; 4 Shanghai Clinical Research Center for Radiation Oncology, Shanghai, China 20032; 5 Shanghai Key Laboratory of Radiation Oncology, Shanghai, China 20032

**Keywords:** Intensity-modulated radiotherapy, IMRT, Radiotherapy, Carcinoma showing thymus-like differentiation, CASTLE

## Abstract

**Background:**

To review our long-term clinical experience, analyze the failure patterns, and give suggestions for target volume delineation of carcinoma showing thymus-like differentiation (CASTLE) treated with intensity-modulated radiotherapy (IMRT).

**Methods:**

From April 2008 to May 2019, 30 patients with CASTLE treated by postoperative or radical IMRT in our center were retrospectively reviewed. A total dose of 56-60 Gy in 28–30 fractions was prescribed to patients without residual disease and 66 Gy in 33 fractions for patients with residual or unresectable disease. Survival rates were calculated using the Kaplan-Meier method. Treatment-related toxicities were graded by National Cancer Institute Common Toxicity Criteria (NCI-CTC) version 4.0.

**Results:**

Among the 30 patients, 12 (40%) received partial resection or biopsy. Lateral lymph node metastasis was observed in 7 (23.3%) patients. During follow-up, regional lymph node recurrence occurred in 2 patients and distant metastasis in 5 patients. With a median follow-up time of 63.5 months, the 5-year local recurrence-free survival (LRFS), regional recurrence-free survival (RRFS), distant metastasis-free survival (DMFS), overall survival (OS) and progression-free survival (PFS) rates were 100, 88.9, 78.9, 93.1 and 78.9%, respectively. For patients with no lateral neck node metastasis, prophylactic radiotherapy for lateral neck nodal regions failed to improve RRFS (p = 0.381) and OS (p = 0.153).

**Conclusion:**

Distant metastasis was the major failure pattern for CASTLE after surgery and IMRT. For patients with no lateral neck node metastasis, the omission of irradiation for lateral neck nodal regions seems to be safe and feasible.

**Supplementary Information:**

The online version contains supplementary material available at 10.1186/s12885-022-10171-9.

## Background

Carcinoma showing thymus-like differentiation (CASTLE) is a rare malignant tumor of the thyroid or adjacent soft tissue in the neck, which accounts for only 0.1–0.15% of all thyroid cancers [1–4]. Until now, fewer than 100 patients have been reported, and most of them are case reports. Optimal treatment for CASTLE remains uncertain. Surgery is generally recommended as the mainstay of treatment [3, 5]. However, due to the lower location and high possibility of extrathyroid invasion, complete resection can be challenging for patients with locally advanced disease [6]. It is reported that the incidence of lymph node metastasis is 50–69% and 60–80% for extrathyroid invasion [5–9]. Therefore, radiotherapy is often used as part of the treatment and the efficiency of radiotherapy has been proved by several studies [6, 7, 10, 11].

However, there is no consensus on the delineation of target volume for CASTLE since very limited data has been published. Accurate target volume delineation is the premise of intensity-modulated radiotherapy (IMRT). Excessive irradiation will increase treatment-related toxicities like dermatitis and neck fibrosis, while insufficiency of target volume may cause tumor recurrence. Hence, in the present study, we reviewed our 11 years of clinical experience, analyzed the failure patterns, and gave suggestions for target volume delineation of CASTLE treated with IMRT. To the best of our knowledge, this is the first study focusing on target volume delineation for this rare disease.

## Methods

### Patients

From April 2008 to May 2019, 30 patients with CASTLE treated by postoperative or radical IMRT in our center were retrospectively reviewed. We collected clinicopathological data, treatment procedures, and clinical outcomes. The patterns of treatment failure were also analyzed. The study was approved by the Institutional Review Board of Fudan University Shanghai Cancer Center. Informed consent was obtained from all patients.

### Intensity-modulate radiotherapy

**Fig. 1:**
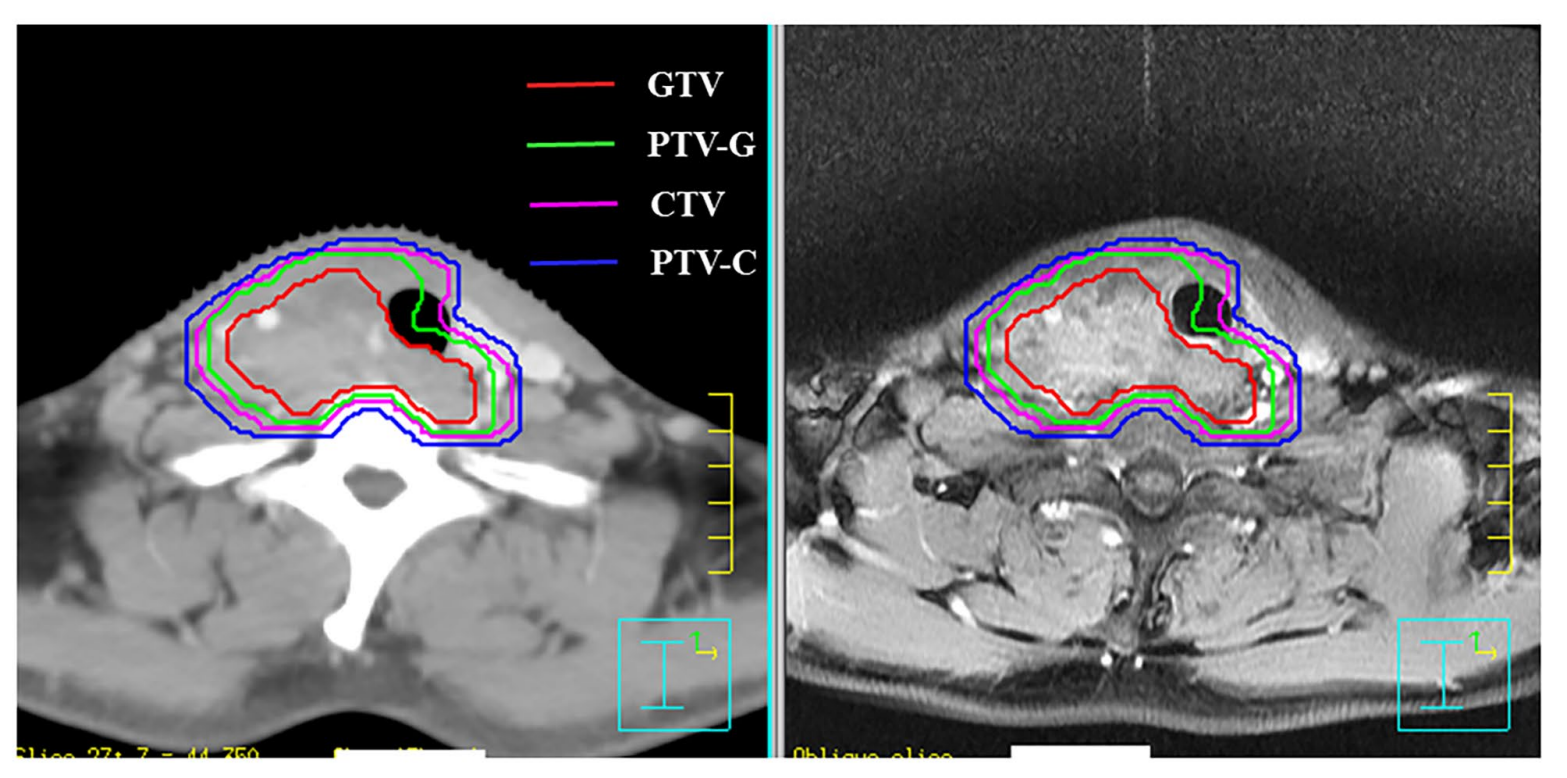
Examples of target volume delineation for carcinoma showing thymus-like differentiation GTV, gross tumor volume; PTV-G, planning target volume for GTV; CTV, clinical target volume; PTV-C, planning target volume for CTV.

IMRT was started 4 to 6 weeks after surgery or early after diagnosis. The techniques of IMRT were detailed in our previously published data [7]. Briefly, a thermoplastic mask of the head and shoulder was used for patient immobilization. Patients received computed tomography (CT) simulation at 5 mm thickness of the head and neck region in the supine position. Image fusion of magnetic resonance imaging (MRI) and CT was recommended for target volume delineation. The gross tumor volume (GTV) was defined as all primary gross tumors and involved lymph nodes determined by imaging and clinical findings. The clinical target volume (CTV) included the GTV or tumor bed plus a 5 to 10 mm margin to encompass any microscopic extension. The planning target volume (PTV) was defined as the CTV plus a 3 to 5 mm margin to encompass setup error. Neck nodal level VI was conventionally included in the CTV. If there was no positive lymph node, neck nodal levels II-V were not included in the CTV. Examples of target delineation were showed in Fig. 1. Positive lymph nodes were defined as follows: (1) pathologically diagnosed; (2) minimum axial diameter ≥ 1 cm; (3) extranodal extension or circular enhancement. The prescribed dose was 60 Gy for patients without residual disease and 66 Gy for patients with residual or unresectable disease. Conventional fractionation (2 Gy per fraction, one fraction per day, five days per week) was used.

### Patient evaluation

Patients were assessed weekly during IMRT. After IMRT, patients were follow-up every three months in the first two years, every six months during the years 3–5, and annually after that. Follow-up assessments after treatment included examination of the neck, thyroid function tests, and ultrasound or MRI for the thyroid and neck. Chest CT scan, and ultrasound or CT of the abdomen were performed every 6–12 months. Additional tests were performed when clinically indicated.

### Definition of failure pattern

The images of MRI or CT scans obtained at the time of recurrence were transferred to the pretreatment planning CT. The dose-volume histogram (DVH) was used to calculate the radiation dose received by the recurrent tumor (GTVrecur) region. The recurrences were classified into five types based on combined spatial and dosimetric criteria [12, 13]:

Type A (central high dose): the mapped centroid of GTVrecur originates in high dose PTV, and ≥ 95% of GTVrecur was within the 95% isodose (high dose PTV);

Type B (peripheral high dose): the mapped centroid of GTVrecur originates in high dose PTV, and < 95% of GTVrecur was within the 95% isodose (high dose PTV);

Type C (central elective dose): the mapped centroid of GTVrecur originates in lower dose PTV, and ≥ 95% of GTVrecur was within the 95% isodose (lower dose PTV);

Type D (peripheral elective dose): the mapped centroid of GTVrecur originates in lower dose PTV, and < 95% of GTVrecur was within the 95% isodose (lower dose PTV);

Type E (extraneous dose): the mapped centroid of GTVrecur originates outside all PTVs.

### Statistical methods

All the statistical analysis was performed using the Statistical Package for Social Sciences (SPSS version 24.0) software. The survival rates were calculated from the day of the first treatment. The Kaplan-Meier method was used to calculate the local recurrence-free survival (LRFS), regional recurrence-free survival (RRFS), distant metastasis-free survival (DMFS), overall survival (OS), and progression-free survival (PFS) rates. Treatment-related toxicities were graded by National Cancer Institute Common Toxicity Criteria (NCI-CTC) version 4.0.

## Results

### Patient characteristics

A total of 30 patients were enrolled in this study. Patients’ characteristics were summarized in Table 1. The median age was 53 years (range 37–61 years). The ratio of male to female was 1:1. Lymph node metastasis was found in 50% of the patients, among which 53.4% were central and 46.6% were lateral. Tumor extension to adjacent organs, including recurrent laryngeal nerve, great vessels, muscles, esophagus, or trachea was found in 21 (70%) patients. About 53% of the patients received complete resection, and 40% received partial resection or biopsy only. Two patients received cisplatin-based concurrent chemotherapy. After treatment, complete remission (CR) was achieved in 26 (86.7%) patients, and partial remission (PR) in 4 (13.3%) patients. Treatment procedures are detailed in Table 2.

**Table 1:** Patient characteristics

	No. (%) of patients
Total	30
Median age (range)	53 (37–61)
Gender	
Male	15 (50)
Female	15 (50)
Tumor location	
Left lobe	18 (60)
Right lobe	11 (36.7)
Isthmus	1 (3.3)
Tumor size (cm)	
≤ 2	3 (10)
2–4	15 (50)
> 4	7 (23.3)
Unknown	5 (16.7)
Lymph node metastasis	
Present	15 (50.0)
Absent	14 (46.6)
Unknown	1 (3.3)
Location of Lymph node(n = 15)	
Lateral	7 (46.6)
Central	8 (53.4)
Tumor extension	
Present	21 (70)
Absent	7 (23.3)
Unknown	2 (6.7)
STE (n = 21)	
RLN	8 (38.1)
Muscles	6 (28.6)
Esophagus	3 (14.3)
Great vessels	4 (19)
Trachea	2 (9.5)
Thymus	1 (4.7)
Parathyroid gland	1 (4.7)

Abbreviations: STE, site of tumor extension; RLN, recurrent laryngeal nerve

**Table 2:** Treatment procedures and clinical outcome

	No. (%) of patients
Type of surgery	
R0	16 (53.3)
R1	2 (6.6)
R2	9 (30)
Biopsy	3 (10)
IMRT dose (Gy)	
Median (range)	60 (56–66)
IMRT duration (days)	
Median (range)	44.5 (39–67)
Systemic therapy	
Yes vs. No	3 (10) vs. 27 (90)
Follow-up time (months)	
Median (range)	63.5 (21–161)
Treatment response	
CR	26 (86.7)
PR	4 (13.3)
Local recurrence	
Present	2 (93.3)
Absent	28 (6.7)
Distant metastasis	
Present	5 (83.3)
Absent	25 (16.7)

Abbreviations: R0, no residual tumor; R1, microscopic residual tumor; R2, macroscopic residual tumor; IMRT, intensity-modulated radiotherapy; CR, complete remission; PR, partial remission

Pathology and immunohistochemical (IHC) data were available for 29 out of 30 patients. Among these, 93.1% (27/29) expressed CD5, and 72.4% (21/29) expressed CD117 totally or partially. Instead, 72.4% (21/29) of the cases were negatively expressed for thyroid transcription factor 1 (TTF-1), which was the marker of thyroid follicular cells.

### Dosimetric data for IMRT

Dose-volume histogram (DVH) statistics were showed in Table 3. The average volume of GTV and CTV were 131.3 cc (21.2-387.2) and 323 cc （92.4-1408.2）, respectively. Rates of dose coverage were excellent for the target volume. The volume receiving less than 95% of the prescribed dose was 0.3% for GTV and 1.2% for CTV. The mean dose was 67.8 Gy for GTV and 62.7 Gy for CTV.

**Table 3:** Dose-volume histograms (DVHs) statistics for IMRT

	GTV Average (range)	CTV Average (range)
Volume (cc)	131.3 (21.2-387.2)	323 (92.4-1408.2)
Maximum dose (Gy)	70.9 (69.2–72.5)	67.6 (63.5–72.5)
Mean dose (Gy)	67.8 (65.9–68.8)	62.7 (57.7–66.8)
Minimum dose (Gy)	56.8 (9.9–65.6)	42.1 (38.2–56.3)
% volume receiving < 95% of the prescribed dose	0.3 (0-1.7)	1.2 (0–12)
% volume receiving ≥ 100% of the prescribed dose	92.1(47.5–100)	94.0(77.6–99.7)
% volume receiving ≥ 110% of the prescribed dose	0	15.8 (0-67.1)

IMRT, intensity-modulated radiotherapy; GTV, gross tumor volume; CTV, clinical target volume

### Survival outcome

**Fig. 2:**
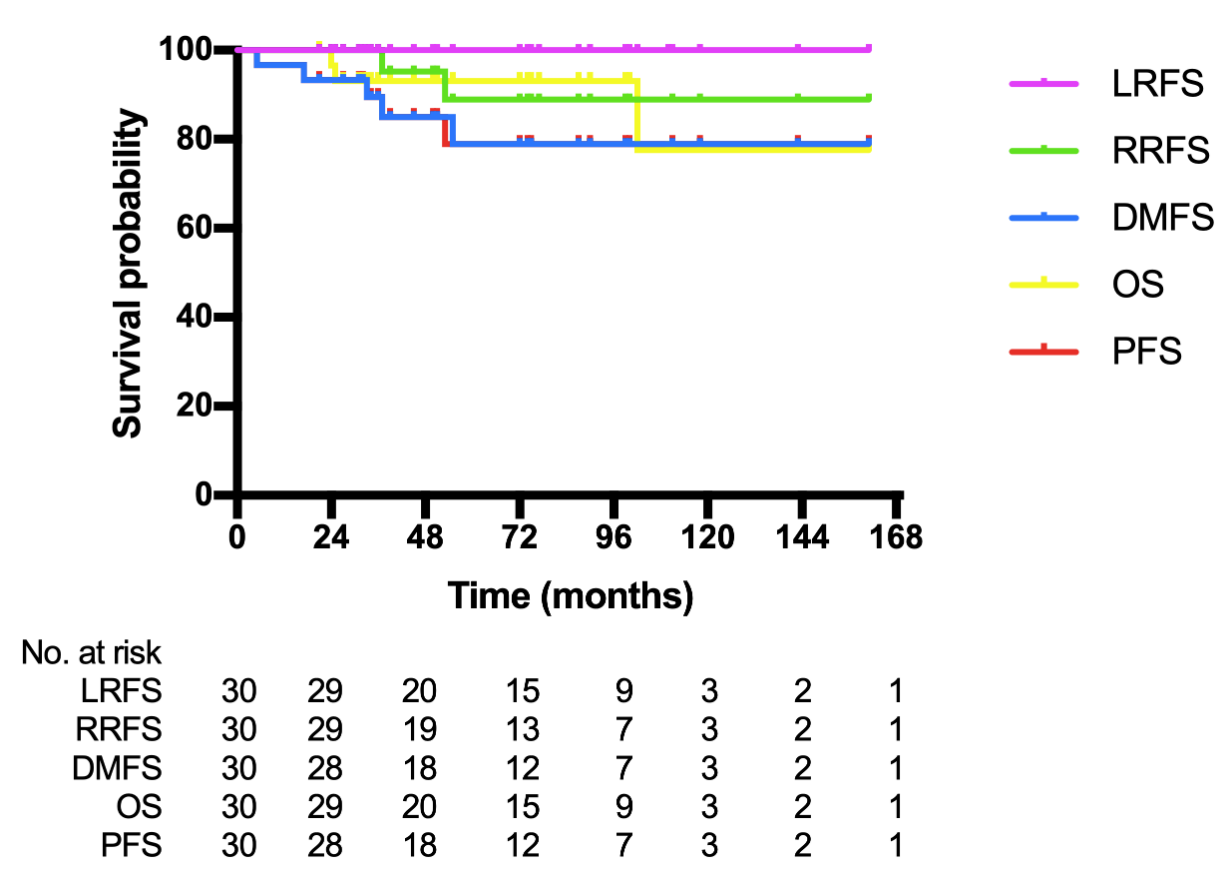
Kaplan-Meier curves showing local recurrence-free survival (LRFS), regional recurrence-free survival (RRFS), distant metastases-free survival (DMFS), overall survival (OS), and progression-free survival (PFS) for patients with carcinoma showing thymus-like differentiation

**Fig. 3:**
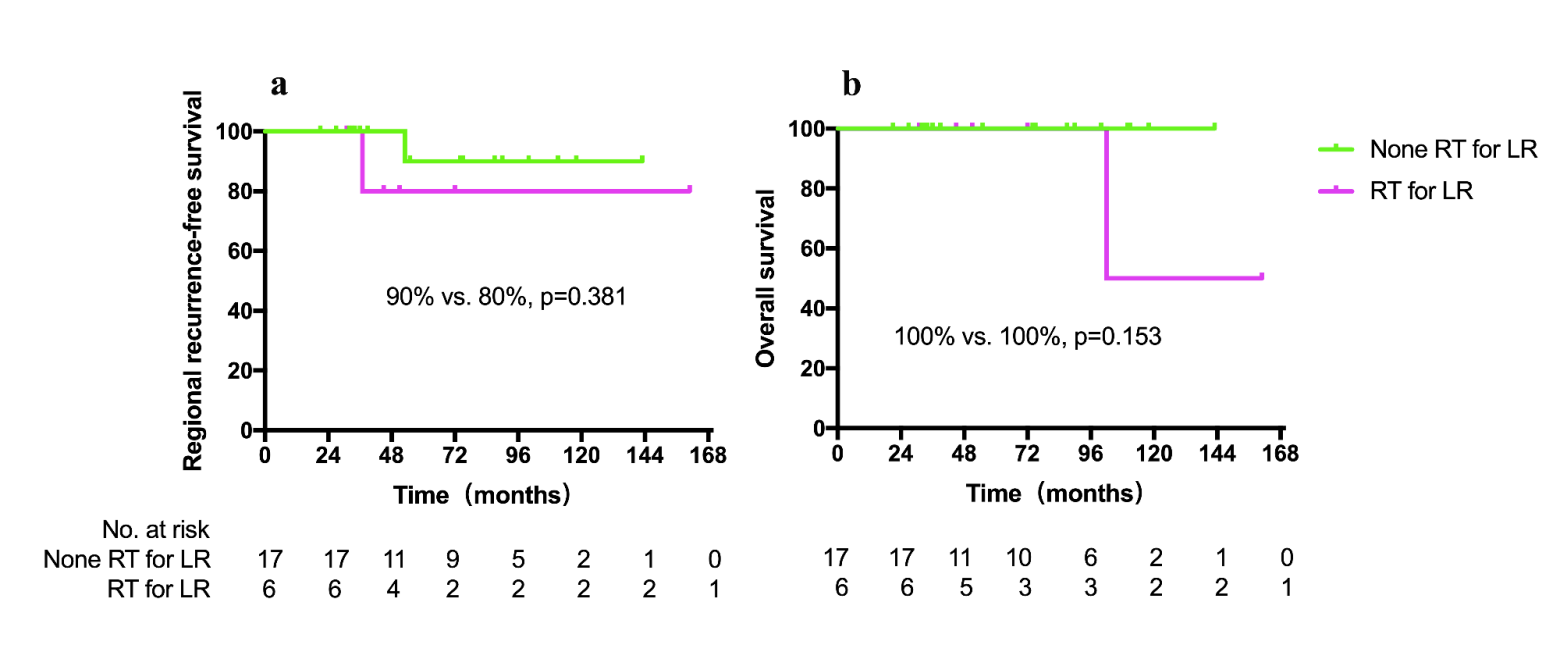
Kaplan-Meier estimate of (a) regional recurrence-free survival (RRFS), and (b) overall survival (OS) between patients with or without irradiation for lateral neck nodal regions RT, radiotherapy; LR: lateral neck nodal regions

The median follow-up time was 63.5 months (range 21–161 months). The 5-year LRFS, RRFS, DMFS, OS, and PFS were 100, 88.9, 78.9, 93.1, and 78.9%, respectively (Fig. 2). In the subgroup analysis, we divided patients with no lateral neck node metastasis (23 patients) into two groups: group A (6 patients) with prophylactic irradiation for lateral neck nodal regions (levels II-V), while group B (17 patients) without prophylactic irradiation for lateral neck nodal regions. The results showed that there was no significant difference in RRFS (p = 0.381) and OS (p = 0.153) between the two groups (Fig. 3). Patient characteristics for groups A and B were shown in Supplementary Table 1.

### Failure patterns and salvage treatment

Two patients had regional lymph node recurrence. All the recurrences were in the contralateral supraclavicular fossa and were defined as type E (extraneous) failures. Distant metastasis was found in 5 patients. Common sites for distant metastasis were lung (3 patients), bone (1 patient), and liver (1 patient). Two patients suffered both regional and distant failure.

Salvage treatment, including surgery, radiotherapy, and systemic therapy, was given to patients with recurrence or distant metastases. If acceptable, clinical trials were the first choice. For patients with oligometastatic or oligoprogressive tumors, SBRT was implemented.

### Treatment complications

The majority of acute toxicities related to IMRT were grade 1–2. Grade 3 mucositis or dermatitis occurred only in 2 patients. No grade 4 toxicities were observed. Details were showed in Table 4. Three patients suffered treatment interruption due to other diseases, including aspiration pneumonia and herpes zoster. After appropriate treatment, they continued and finished IMRT. There was no late toxicity (≥ grade 2) related to IMRT, such as radiation pneumonitis, neck fibrosis, or esophageal stenosis.

**Table 4:** Acute toxicities during IMRT

Toxicities	No. of patients by toxicity grade (%)				
	Grade 1	Grade 2	Grade 3	Grade 4	Grade 5
Mucositis	9 (30)	20 (66.7)	1 (3.3)	0	0
Dermatitis	25 (83.3)	4 (13.3)	1 (3.3)	0	0
Skin pigmentation	26 (86.7)	0	0	0	0
Anemia	4 (13.3)	1 (3.3)	0	0	0
Leukopenia	5 (16.7)	1 (3.3)	0	0	0
Thrombocytopenia	3 (10)	0	0	0	0

IMRT, intensity-modulated radiotherapy

### Prognostic factors

Univariate analysis, taking age, gender, tumor size, tumor extension, surgery type, and lymph node metastasis as prognostic factors for RRFS, DMFS, OS, and PFS, was performed. Univariate analysis showed that surgery type (p = 0.026, 0.000, 0.026) and lateral neck node metastasis (p = 0.024, 0.009, 0.024) were potential prognostic factors for DMFS, OS and PFS (Supplementary Table 2). However, the multivariate analysis failed to further validate the differences (Supplementary Table 3). We attributed it to the relatively small sample size and few end-point events.

## Discussion

CASTLE is an extremely rare malignant tumor, which is thought to arise from ectopic thymus or branchial pouch remnants [14]. The morphological, immunohistochemical, and molecular features of CASTLE are similar to those of thymic carcinoma. The carcinogenesis of CASTLE is the result of combined actions by a series of oncogenes and tumor suppressors. The study from Wang et al. [15] indicated that abnormal expression of p16, Bcl-2, p53, E-cadherin, C-KIT, CA-IX, EGFR, and HER-2 might play a role in the tumorigenesis and development of CASTLE. Patients with HER-2 overexpression showed a worse prognosis, suggesting that HER-2 overexpression may enhance the invasive and metastatic potential of CASTLE. However, the molecular pathological mechanism of CASTLE is still unclear. Further studies are needed to explore the molecular networks of this rare disease.

Although the optimal treatment modality for CASTLE remains uncertain, an increasing number of studies have indicated the important role of radiotherapy in treating CASTLE. Due to the high possibility of extrathyroid invasion and high incidence of lymph node metastasis, adjuvant radiotherapy is often used as part of the treatment [5–9]. And growing evidence has validated the efficiency of adjuvant radiotherapy in recent years [5–7, 10, 11]. Choi et al. [10] reported that adjuvant radiotherapy reduced about 43% of the recurrence for patients with positive lymph nodes. In the study by Gao et al. [6], the median survival time was significantly longer in the surgery and adjuvant radiotherapy group than in the surgery alone group (17.1 vs. 8.8 years, p = 0.034). Moreover, radiotherapy can also play an essential role in tumor control for patients with unresectable disease. Petra et al. [16] recently reported a patient with locally advanced disease which was not suitable for surgery and thus underwent radical IMRT alone. Exciting complete remission was achieved after IMRT at a dose of 70 Gy. In the present study, palliative resection was administered to one of the patients due to the tumor invasion of adjacent great vessels. Then the patient received radical IMRT at a dose of 66 Gy in 33 fractions. Partial remission was achieved. Seven years after IMRT, the patient is still alive with stable disease at our last follow-up. All the above implies the indispensable role of radiotherapy in treating CASTLE.

There is no consensus on target volume delineation for CASTLE. Most of the previous studies reported the radiation dose only and with no specification of the target volume. To the best of our knowledge, this is the first study to explore the proper target volume delineation for this rare disease. As we know, neck nodal level VI is the sentinal node for thyroid tumors. Excessive irradiation for the neck can induce dermatitis, especially for the lower neck (as most of the CASTLE arises in the lower part of the thyroid lobe). Severe dermatitis may cause the interruption of radiotherapy and influence the treatment effect. After treatment, it may also cause neck fibrosis, which will seriously affect patients’ long-term quality of life. Hence, in the present study, we compared the effects of prophylactic irradiation and omitting irradiation to lateral neck regions on the survival rates of patients with no lateral neck node metastasis. The results showed that there was no significant difference in RRFS and OS between the two groups, which suggested that it was safe and feasible to omit irradiation to lateral neck regions for patients with no lateral neck node metastasis. Of course, there may be a certain deviation due to the small sample size and few end-point events. Prospective studies are warranted to further verify our conclusion.

Two patients had lymph node recurrence, which occurred in the contralateral supraclavicular fossa. Both of the patients underwent R0 resection at first treatment. One of the patients (Pt.1) had positive lymph nodes in level VI and the other patient (Pt.2) had negative lymph node. Pt.1 received adjuvant RT to the tumor bed and neck levels III, IV, and VI. Pt.2 received RT for tumor bed and neck level IV. Recurrences occurred 3 and 5 years respectively after surgery. It’s worth noting that both of the patients suffered synchronous or metachronous lung and mediastinal lymph node metastasis. It seems that the recurrence of the supraclavicular lymph node may be caused by lung and mediastinal lymph node metastasis.

Distant metastasis is the major failure pattern for CASTLE after surgery and IMRT. However, the role of chemotherapy remains unclear because of the rarity of the disease. Different regimens have been explored, including cisplatin, epirubicin, docetaxel, irinotecan, vincristine, and cyclophosphamide. But the results seem to be heterogeneous. Hanamura et al. [17] reported a good response to platinum-based chemotherapy of a patient with lung metastasis from CASTLE. The author suggested that CASTLE is a chemosensitive tumor, and chemotherapy should be recommended for patients with advanced or metastatic disease. However, in contrast to Hanamura’s study, Roka et al. [18] found no response to three different regimens of a patient with liver metastasis. In the current study, one patient with gross residual disease after surgery received concurrent chemotherapy (docetaxel and cisplatin). However, the chemotherapy was stopped because of the toxicities after one cycle. CR was achieved for the positive lymph node and PR for the primary tumor after IMRT. Unfortunately, the patient suffered lung metastasis 18 months after IMRT. Systematic therapy combined with IMRT may be an effective treatment option for patients with locally advanced diseases. However, the optimal regimen and combination mode needs to be further investigated.

Immunotherapy is a revolutionary breakthrough in the treatment of cancer. The effectiveness and safeness have been proved in different tumors in the past few years. As to CASTLE, Lorenz et al. [19] reported the first case in 2019. The patient suffered multiple metastatic diseases (lung, mediastinal, hilar, and upper mesenteric lymph nodes and pleura) 10 years after treatment of CASTLE in the parotid gland. The tumor showed a high expression level of PD-L1 (60%), and the PD-L1 inhibitor (pembrolizumab 200 mg every three weeks) was given to the patient. Partial remission was achieved four months after the start of immunotherapy, and treatment-related toxicities were mild and tolerable. The result was exciting. Further research on immunotherapy with or without chemotherapy or radiotherapy for patients with advanced or metastatic CASTLE is warranted.

## Conclusion

Our long-term results showed that surgery combined with IMRT is an effective treatment for patients with CASTLE. Distant metastasis is the major failure pattern. For patients with no lateral neck node metastasis, the omission of irradiation for lateral neck nodal regions seems to be safe and feasible. Further prospective research is warranted.

## Electronic supplementary material

Below is the link to the electronic supplementary material.

Supplementary Material 1

## Data Availability

The datasets used and/or analyzed during the current study are available fromthe corresponding author on reasonable request
