# Supplementary material for: Failure pattern and suggestions for target volume delineation of carcinoma showing thymus-like differentiation treated with intensity-modulated radiotherapy
Source: BMC Cancer. 2022 Oct 21;22:1083. doi: 10.1186/s12885-022-10171-9 (PMC9585782; doi:10.1186/s12885-022-10171-9)
Supplement: Supplementary file 1 — Supplementary Material 1 [file 12885_2022_10171_MOESM1_ESM.docx]

**Supplementary Table 1**. Characteristics for patients with or without prophylactic irradiation for lateral neck nodal regions

|  | No. (%) of patients | |
| --- | --- | --- |
|  | Group A^*^ | Group B^**^ |
| Total number | 6 | 17 |
| Tumor size (cm) |  |  |
| ≤4cm | 3（50） | 11（64.7） |
| ＜4cm | 1（16.7） | 4（23.5） |
| Level VI LN(+) |  |  |
| Yes | 1（16.7） | 6（35.3） |
| No | 5（83.3） | 11（64.7） |
| Tumor extension |  |  |
| Present | 3（50） | 12（70.6） |
| Absent | 2（33.3） | 4（23.5） |

Abbreviations: LN(+), positive lymph node.

^*^Group A : Patients with prophylactic irradiation for lateral neck nodal regions (levels II-V);

^**^Group B: Patients without prophylactic irradiation for lateral neck nodal regions.

**Supplementary Table 2 .** Impact of prognostic factors on treatment results by univariate analysis.

| Items | 5y-RRFS | | 5y-DMFS | | 5y-OS | | 5y-PFS | |
| --- | --- | --- | --- | --- | --- | --- | --- | --- |
|  | % | P | % | P | % | P | % | P |
| Age(yr) |  |  |  |  |  |  |  |  |
| <60 | 88.2 | 0.728 | 80.5 | 0.275 | 76.9 | 0.228 | 80.5 | 0.694 |
| ≥60 | 100 |  | 66.7 |  | 66.7 |  | 66.7 |  |
| Gender |  |  |  |  |  |  |  |  |
| Male | 90.9 | 0.935 | 78.8 | 0.694 | 85.7 | 0.073 | 78.8 | 0.275 |
| Female | 85.7 |  | 76.9 |  | 100 |  | 76.9 |  |
| Tumor size (cm) |  |  |  |  |  |  |  |  |
| ≤2 | 100 | 0.650 | 100 | 0.633 | 100 | 0.243 | 100 | 0.633 |
| 2-4 | 81.5 |  | 73.4 |  | 100 |  | 73.4 |  |
| >4 | 100 |  | 71.4 |  | 71.4 |  | 71.4 |  |
| Tumor extension |  |  |  |  |  |  |  |  |
| Present | 100 | 0.056 | 84.8 | 0.988 | 90.0 | 0.397 | 84.8 | 0.988 |
| Absent | 66.7 |  | 66.7 |  | 100 |  | 66.7 |  |
| Type of surgery |  |  |  |  |  |  |  |  |
| R0 | 79.5 | 0.618 | 79.5 | ***0.026*** | 100 | ***0.000*** | 79.5 | ***0.026*** |
| R1 | 100 |  | 100 |  | 100 |  | 100 |  |
| R2 | 100 |  | 87.5 |  | 100 |  | 87.5 |  |
| Biopsy | 100 |  | 33.3 |  | 33.3 |  | 33.3 |  |
| LN Metastasis |  |  |  |  |  |  |  |  |
| Present | 90.9 | 0.989 | 70.7 | 0.137 | 85.7 | ***0.018*** | 70.7 | 0.137 |
| Absent | 87.5 |  | 87.5 |  | 100 |  | 87.5 |  |
| Positive lateral LN |  |  |  |  |  |  |  |  |
| Present | 100 | 0.453 | 57.1 | ***0.024*** | 71.4 | ***0.009*** | 57.1 | ***0.024*** |
| Absent | 85.9 |  | 85.9 |  | 100 |  | 85.9 |  |

Abbreviations:RRFS, regional recurrence-free survival; DMFS, distant metastasis-free survival; OS, overall survival; and PFS, progression failure-free survival; LN, lymph node

**Supplementary Table 3**. Impact of prognostic factors on treatment results by multivariate analysis (p value).

| Factors | 5y-RRFS | 5y-DMFS | 5y-OS | 5y-PFS |
| --- | --- | --- | --- | --- |
| Age(years) |  |  |  |  |
| <60 vs. ≥60 | 0.983 | 0.617 | 0.817 | 0.617 |
| Gender |  |  |  |  |
| Male vs. Female | 0.821 | 0.745 | 0.978 | 0.745 |
| Tumor size (cm) |  |  |  |  |
| ≤2 vs. 2-4 vs. ≥4 | 0.940 | 0.905 | 0.995 | 0.905 |
| Tumor extension |  |  |  |  |
| Present vs. Absent | 0.985 | 0.805 | 0.924 | 0.805 |
| Type of surgery |  |  |  |  |
| R0 vs. R1 vs. R2 vs. Biopsy | 0.964 | 0.948 | 0.971 | 0.948 |
| LN Metastasis |  |  |  |  |
| Present vs. Absent | 0.994 | 0.936 | 0.980 | 0.936 |
| Positive lateral LN |  |  |  |  |
| Present vs. Absent | 0.984 | 0.736 | 0.852 | 0.736 |

Abbreviations: RRFS, regional recurrence-free survival; DMFS, distant metastasis-free survival; OS, overall survival; and PFS, progression failure-free survival; LN, lymph node
